# Glutathione and Xanthine Metabolic Changes in Tamoxifen Resistant Breast Cancer Cell Lines are Mediated by Down-Regulation of *GSS* and *XDH* and Correlated to Poor Prognosis

**DOI:** 10.7150/jca.96659

**Published:** 2024-05-30

**Authors:** Mohammad Alwahsh, Yazan Hamadneh, Rosemarie Marchan, Lina A. Dahabiyeh, Ala A Alhusban, Aya Hasan, Jawad Alrawabdeh, Roland Hergenröder, Lama Hamadneh

**Affiliations:** 1 Faculty of Pharmacy, Al-Zaytoonah University of Jordan, Amman-17138, Jordan.; 2 School of Medicine, The University of Jordan, Amman, Jordan.; 3 Leibniz Research Centre for Working Environment and Human Factors at the TU Dortmund (IfADo), Ardeystrasse 67, 44139 Dortmund, Germany.; 4 Department of Pharmaceutical Sciences, School of Pharmacy, The University of Jordan, 11942 Amman, Jordan.; 5 Leibniz-Institut für Analytische Wissenschaften-ISAS-e.V., 44139 Dortmund, Germany.; 6 Department of Basic Medical Sciences, Faculty of Medicine, Al-Balqa Applied University, 19117, Al-Salt, Jordan.

**Keywords:** metabolomics, xanthine, glutathione, tamoxifen resistance, xanthine dehydrogenase

## Abstract

**Background:** Tamoxifen is commonly used in the treatment of hormonal-positive breast cancer. However, 30%-40% of tumors treated with tamoxifen develop resistance; therefore, an important step to overcome this resistance is to understand the underlying molecular and metabolic mechanisms. In the present work, we used metabolic profiling to determine potential biomarkers of tamoxifen resistance, and gene expression levels of enzymes important to these metabolites and then correlated the expression to the survival of patients receiving tamoxifen.

**Methods:** Tamoxifen-resistant cell lines previously developed and characterized in our laboratory were metabolically profiled with nuclear magnetic resonance spectroscopy (NMR) using cryogenic probe, and the findings were correlated with the expression of genes that encode the key enzymes of the significant metabolites. Moreover, the effect of significantly altered genes on the overall survival of patients was assessed using the Kaplan-Meier plotter web tool.

**Results:** We observed a significant increase in the levels of glutamine, taurine, glutathione, and xanthine, and a significant decrease in the branched-chain amino acids, valine, and isoleucine, as well as glutamate and cysteine in the tamoxifen-resistant cells compared to tamoxifen sensitive cells. Moreover, xanthine dehydrogenase and glutathione synthase gene expression were downregulated, whereas glucose-6-phosphate dehydrogenase was upregulated compared to control. Additionally, increased expression of xanthine dehydrogenase was associated with a better outcome for breast cancer patients.

**Conclusion:** Overall, this study sheds light on metabolic pathways that are dysregulated in tamoxifen-resistant cell lines and the potential role of each of these pathways in the development of resistance.

## Introduction

Breast cancer is the most diagnosed cancer worldwide, with an estimated 2.3 million new cases in 2020 1. It is also the leading cause of cancer-related deaths in females in countries with low/medium human development index (HDI); whereas, it is surpassed only by lung cancer in high HDI countries 1. There are four clinically relevant molecular subtypes of invasive ductal carcinoma: luminal A, luminal B, HER2/neu, and basal-like breast cancer, based on the expression of the hormone receptors: estrogen (ER) and progesterone (PR), as well as the human epidermal growth factor (HER2). Luminal A is the most common molecular subtype of breast cancer, accounting for approximately 50% of invasive breast cancer cases 2, and is characterized as estrogen receptor-positive (ER+), progesterone receptor-positive (PR+), HER2/Neu-negative, with low Ki-67 expression 3. Abnormal estrogen signaling through ERα is a major driver of tumorigenesis in luminal A and luminal B breast cancers 4, as it promotes the proliferation of cancer cells by overexpressing cyclin D1 and c-Myc, both of which enable the tumors to bypass the G1/S checkpoint 5.

Selective estrogen receptor modulators (SERMs) are a class of compounds that inhibit the estrogen signaling pathway and cell cycle progression, which consequently inhibits cell growth 6. Tamoxifen is the prototypical SERM that has both agonistic and antagonistic effects depending on the tissue, for example in breast tissue, tamoxifen exhibits an antagonistic effect 7. Breast cancer treatment is usually multimodal, utilizing surgery, radiotherapy, chemotherapy, immunotherapy, and targeted therapy. Most patients undergo surgery combined with adjuvant and/or neoadjuvant chemotherapy, depending on the stage of the disease, followed by a long course of SERM if the cancer cells express hormonal receptors. Patients with the luminal A subtype are usually treated with tamoxifen for 5 to 10 years 8. However, 15% to 20% of ER+ tumors are intrinsically resistant to endocrine treatment while 30% to 40% of ER+ tumors develop endocrine resistance to this class of drugs throughout treatment 9, 10. Therefore, understanding the underlying mechanisms of tamoxifen resistance in breast cancer is critical to reducing the prevalence of treatment resistance in breast cancer and associated mortality.

Metabolic reprogramming; a hallmark of cancer and treatment resistance development seen in tumors including those of the breast is driven by genetic and epigenetic factors to support their continuous growth and survival in harsh environments 11, 12. Most of these changes modulate amino acids, lipids, and glucose metabolism 13. The first identified and widely recognized metabolic reprogramming pathway is the Warburg effect - a phenomenon that involves the increase in glucose consumption rate and its fermentation to lactate in tumors, even in the presence of oxygen 14, 15. In addition to glucose, an increased influx of glutamine into cells has been linked to the development of resistance to chemotherapy and endocrine therapies by activating pathways that support both survival and proliferation 16. This discovery paved the way for the identification of potential therapeutic targets and resistance markers that could significantly contribute to the treatment of breast cancer 17, 18.

Metabolomics is an advanced analytical approach used to profile the complete set of metabolites, such as small molecule products or intermediates of biochemical processes inside cells, tissues, organs, systems, or organisms 19. It is one of the four branches of omics sciences alongside genomics, transcriptomics, and proteomics that altogether aim to comprehensively investigate cellular components at different levels 20. In this study, the metabolomic profiles of three tamoxifen-resistant MCF-7 cell lines were characterized using nuclear magnetic resonance (NMR) spectroscopy. Additionally, changes in the expression levels of genes that are associated with the significantly altered metabolites and their respective metabolic pathways were studied using RT-qPCR and correlated with alterations in the metabolite levels, followed by correlation to the survival of patients receiving tamoxifen using data obtained from Kaplan Meier webtool. The findings in the present work contribute to understanding the metabolic and molecular mechanisms behind tamoxifen resistance in breast cancer.

## Materials and Methods

### Cell culturing and tamoxifen-resistance development

MCF-7 cell lines (ATCC, USA) were cultured in RPMI 1640 media (EuroClone S.p.A., Italy) supplemented with 1% penicillin-streptomycin, 1% L-Glutamine, and 10% fetal bovine serum (FBS) and incubated in a 37 °C incubator under 5% CO2 atmosphere. Three tamoxifen-resistant MCF7 cell lines were developed using 3 different tamoxifen concentration approaches as previously described 21-23. The first approach involved treating the cells with increasing tamoxifen concentrations starting with 100 nM until a concentration of 50 µM was reached. In the second approach, the cells were treated with a starting concentration of 100 nM and increased until 35 µM was reached. The cells were then treated six more times with 35 µM. For each media change with a new concentration, cells were treated with tamoxifen for 24 h, then the media was replaced with fresh media. The next dose was added when cells reached 70% confluence. In the third approach, cells were treated with a starting concentration of 100 nM, and the concentration was increased until reaching 35 µM, the treatment was then fixed at 35 µM, which was repeated four times. The cells were then permanently maintained in media containing only 1 µM of tamoxifen as seen in **(Supplementary Scheme 1)**. The continued treatment with 1 µM was done as this concentration is within range of the serum concentrations of the sum of tamoxifen and its metabolites in breast cancer patients treated with tamoxifen 24. Cells were labeled 50, 35x6, and 4+1 (each group contains 9 samples) to represent the concentration and number of treatments the cells received throughout the metabolic and molecular studies.

### Metabolites extraction

MCF-7 cells were grown in 100 mm* 20 mm cell culture dishes (Corning, USA) until 80-90% confluency. The media was then removed, and the cells were washed using a prewarmed 37 °C phosphate buffer solution. To quench the metabolism, 500 µl of precooled -48 °C 100% methanol (Sigma, Germany) was added to the cells, which were then incubated for 30 minutes at -80 °C. The cells were then placed on dry ice and scraped, and the suspension was collected in a 2 ml Eppendorf tube. 500 µl of chloroform was added to the cells and the suspension was vortexed for 1 hour at 4 °C. This was followed by the addition of 300 µl of water and the resulting suspension was centrifuged at 18,500 g for 10 minutes at 4 °C. After centrifuging, the upper phase, which mainly contains polar metabolites, was transferred into a new 2 ml Eppendorf and the solvent was evaporated by a vacuum evaporator. Before NMR analysis, the sample was reconstituted in 50 µl of precooled 4 °C methanol, centrifuged at 18,500 g for 10 minutes at 4 °C and the resulting supernatant was transferred into a precooled 2 ml Eppendorf and then stored at -80 °C 25.

### Sample preparation and measurement conditions for NMR

All the NMR experiments were carried out at 600.13 MHz for 1H detection, on BRUKER AVANCE NEO 600 spectrometer equipped with a cryogenic NMR probe to enhance the sensitivity. To achieve water suppression of water signal, cell culture samples were measured with a double watergate sequence with excitation sculpting 26 dissolved in D2O. The “Electronic Reference To access in vivo Concentrations” (ERETIC) technique was employed for the calibration of the sample spectra 27.

### NMR-related statistical analysis

The web server Metaboanalyst 5.0 was used to conduct univariate and multivariate statistical analyses 28, 29. To prevent the contribution of dilution effects, metabolite data were mean-centered and the intensities of the spectral peaks of each given sample were normalized to the sum of all metabolite concentrations. The boxplots of metabolite concentrations were created using the "geom_boxplot" and "facet_wrap" modules of R studio software.

### NMR data analysis

Chenomx NMR suite 9.0 (Chenomx Inc., Edmonton, AB, Canada) was used for metabolite detection and quantification. Metabolite concentrations were determined using DSS as a reference compound and reported in μM. We also used MetaboAnalyst v5.0 (Xia Lab @ McGill University, Montreal, QC, Canada) 30 to identify the metabolites that contribute to group separation using Partial Least Squares-Discriminant Analysis (PLS-DA), heat map, and pathway analysis. Model robustness was assessed using Receiver Operating Characteristic - Area Under Curve (ROC-AUC) analysis in MetaboAnalyst software. Statistical significance was set at p<0.05 (estimated based on t-test and one-way ANOVA). The metabolite concentration boxplots were created using the R studio software's "geom_boxplot" and "facet_wrap" modules.

### Gene expression assay

Total RNA samples were extracted from tamoxifen resistance and tamoxifen sensitive cells using RNeasy® Plus Mini Kit (QIAGEN, USA). mRNA samples were converted to cDNA using the High-Capacity cDNA Reverse Transcription Kit (Applied Biosystems™, USA). qRT-PCR CFX96 real-time PCR (Bio-rad, USA) and SYBR® Green Master Mix (Bio-rad, USA) were used in gene expression analysis experiments with different primers sets of the genes encoding the primary enzymes involved in the metabolites found to be dysregulated **(Supplementary Table 1)**.

### Kaplan-Meier Plotter

The potential correlation of gene expression on patients' overall survival was assessed using Kaplan-Meier Plotter (www.kmplot.com) 31 among ER/PR positive breast cancer patients receiving tamoxifen as endocrine therapy. Kaplan-Meier Plotter is an open accessed database that allows the correlation of gene expression and survival of thousands of patients having different types of tumors including breast cancer. Expressed genes with p < 0.05 were considered significant and hazard ratios (HR) > 1 of significantly expressed genes were linked to poor prognosis and survival among breast cancer patients.

## Results

### Metabolite profiling of the three tamoxifen-treated groups vs. control group

**Figure 1:**
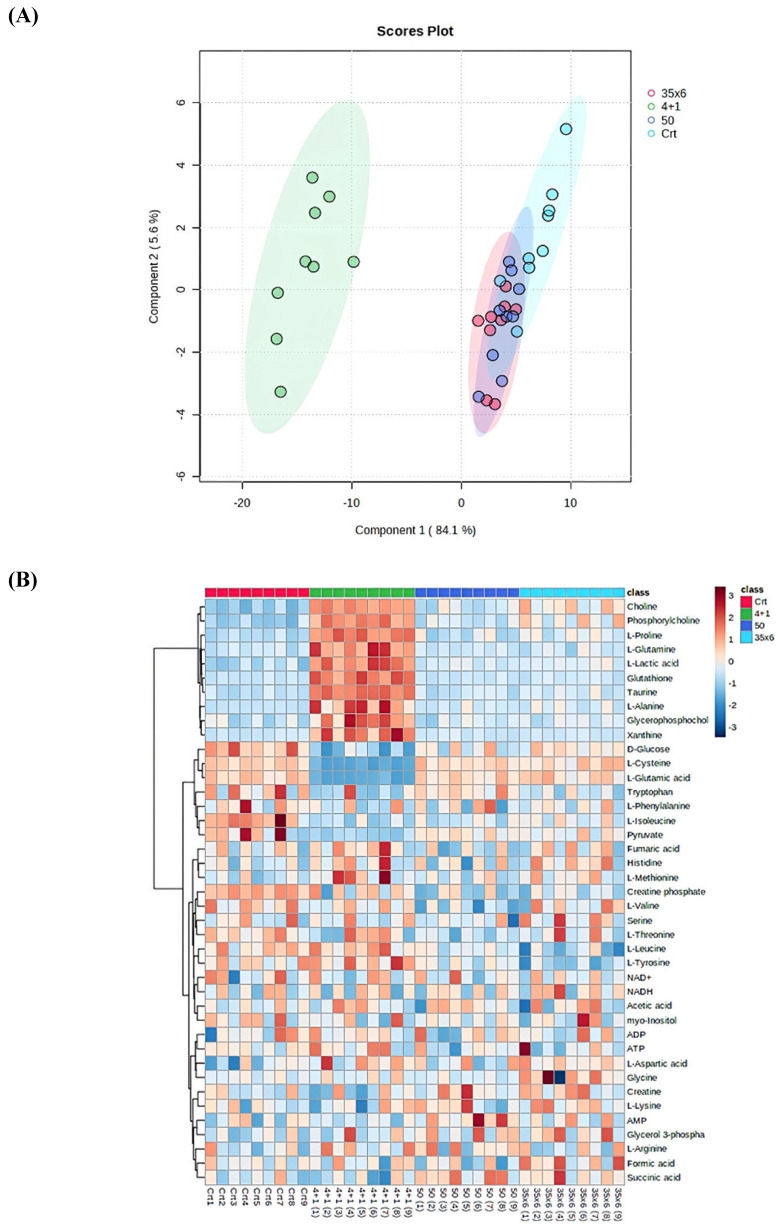
Metabolic profile displayed differential metabolic features in 3 resistance groups (4+1, 50, and 35x6) compared to control by using cryogenic probe NMR spectroscopy. (A**)** PLS-DA score plot as multivariate data analyses based on metabolomics data between three tamoxifen-treated groups (each group contains 9 samples) compared to the control group (n = 9). (B) Heat map presenting the 41 metabolites found in the four treatment groups. The higher values (red) reflect higher metabolite concentrations, and lower values (blue) reflect lower levels.

**Figure 2:**
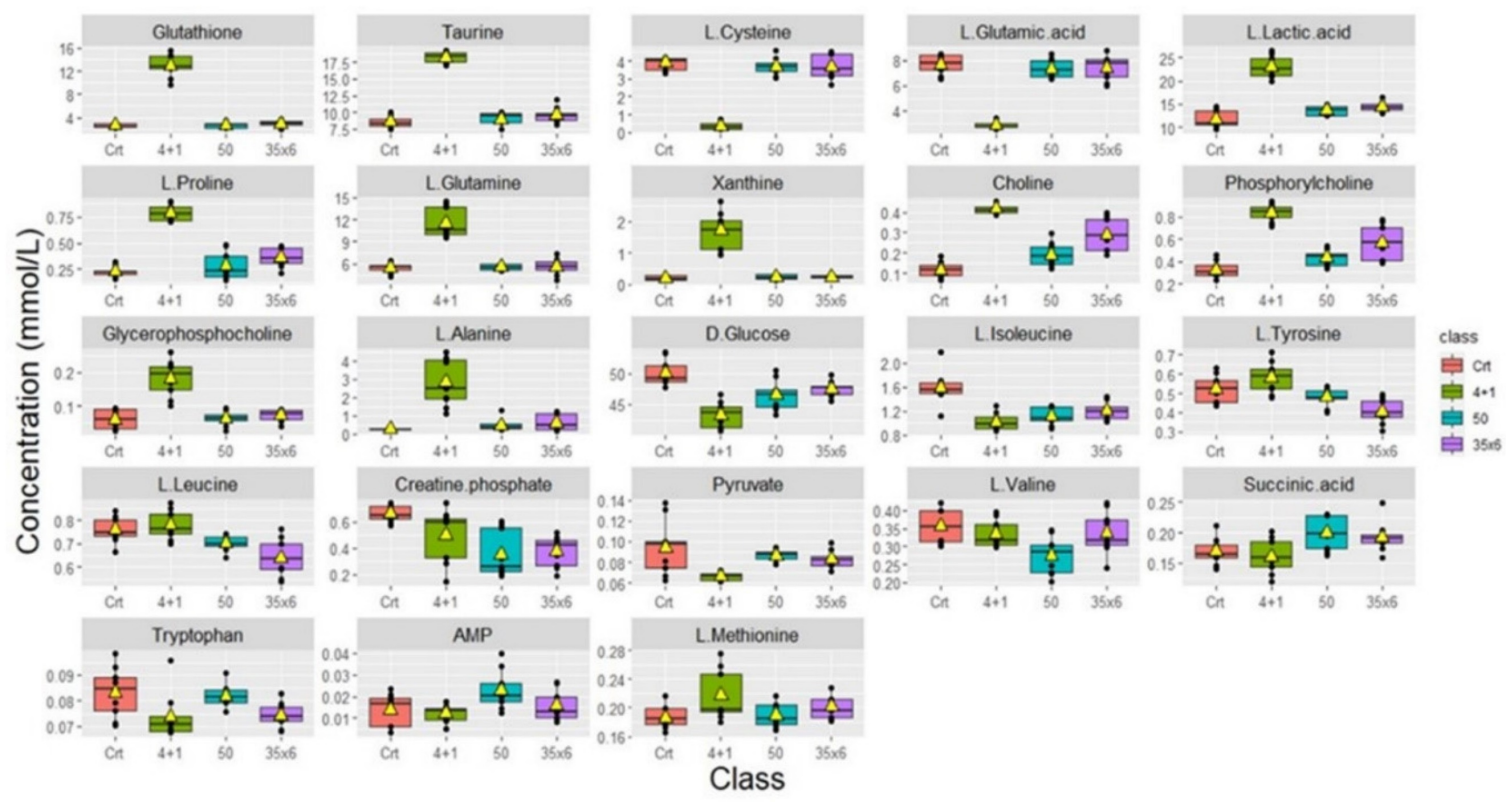
** Metabolic changes in tamoxifen-resistant MCF7 breast cancer cells**. Boxplots showing the concentrations (y-axis) of the significantly altered metabolites in control (n = 9), compared to 50 (n = 9), 35x6 (n = 9), and 4+1 (n = 9) treatment groups analyzed by one-way ANOVA, p-value <0.05. The black bars show the respective median of a distribution, while the yellow triangles show the respective average. Please note that the scale of the y-axis was adapted to the concentration range and is therefore different among the different metabolites.

NMR-based metabolic profiling was used to identify the metabolic properties of four cell lines: control MCF7 cells, which are tamoxifen-sensitive, and three tamoxifen-resistant MCF7 cell lines, by performing univariate and multivariate data analyses. 41 metabolites were detected in the four groups **(Supplementary Figure 1)**. The PLS-DA and heat map in **Figure 1** show that resistant cells treated continuously with 1 µM tamoxifen (4+1), a concentration that is often measured in patients (24), showed different metabolic clusters compared to the other two resistant groups (50 and 35x6) and the control (tamoxifen-sensitive) group, indicating that they had markedly different metabolic characteristics (**Figure 1A**). Furthermore, the concentration of several metabolites was higher in the 4+1 group compared to the other two treated and control groups except for L-cysteine, D-glucose, L-glutamic acid, and pyruvate, which were lower (**Figure 1B and Figure 2**)**.** Of note, the concentration of these four metabolites in the 50 and 35x6 resistant groups was similar to those in the control group (**Figure 1B and Figure 2**).

Boxplots were used to show the concentrations of the most significantly altered metabolites in the treated groups (50), (35x6), and (4+1) compared to the control group (**Figure 2**), with their respective p-values **(Table 1)**. We identified a total of 18 metabolites that are so-called marked metabolites for the 4+1 group **(Table 1, Supplementary Figure 2)**, including taurine, L-glutamic acid, glutathione, L-glutamine, and xanthine. In contrast, 11 metabolites, including L-threonine, L-valine, and choline, are marked metabolites for group 50 **(Table 1, Supplementary Figure 3)**. Finally, L-tyrosine, L-leucine, L-alanine, and L-aspartic acid are among the 14 marked metabolites for group (35x6)** (Table 1, Supplementary Figure 4)**.

According to univariate and multivariate data analyses (**Figures 1 and 2**), resistant cells treated continuously with 1 µM tamoxifen (4+1), showed a distinct metabolic profile compared to the other two resistant groups and the control group.

### Metabolite profiling of cells treated continuously with tamoxifen (4+1) vs. control untreated cells

**Figure 3:**
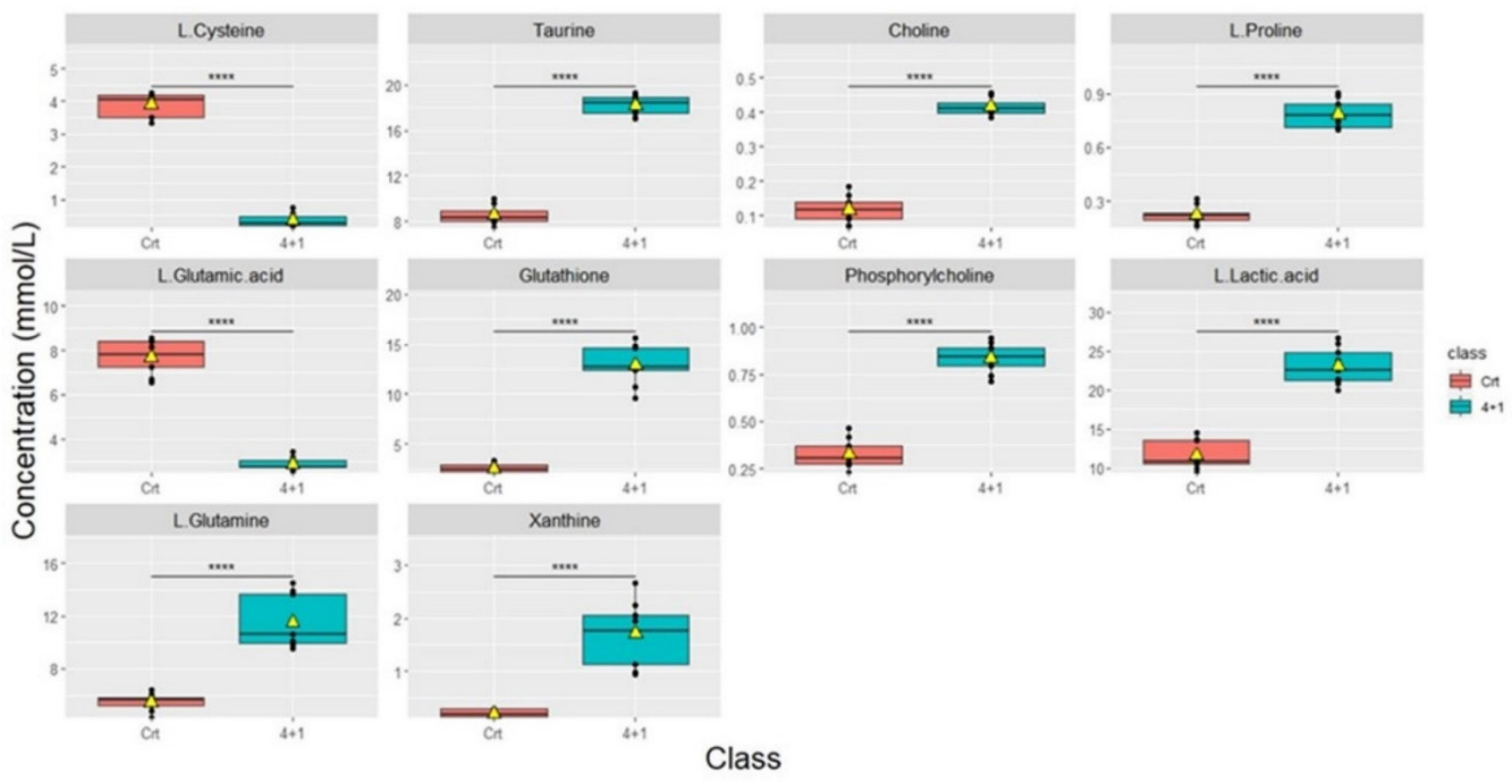
Boxplots showing the concentrations (y-axis) of the most significantly altered metabolites found in 4+1 vs control groups. The black bars show the respective median of the distribution, while the yellow triangles show the respective average. The scale of the y-axis was adapted to the concentration range and is therefore different among the different metabolites. Boxplots are representative of n = 9 replicates. **** represents *p* < 0.0001 calculated using the student's t-test.

Significant differences in metabolic concentrations were discovered in the 4+1 group when compared to the control. As described above, with the respective p-values provided (**Table 1**). The top 10 significantly altered metabolites between the control and the 4+1 treatment group are taurine, choline, L-proline, glutathione, phosphorylcholine, L-lactic acid, L-glutamine, and xanthine, which were all significantly higher in 4+1 group compared to the control group (**Figure 3**). In contrast, L-cysteine and L-glutamic acid were found to be significantly decreased in the 4+1 group compared with control.

**Figure 4:**
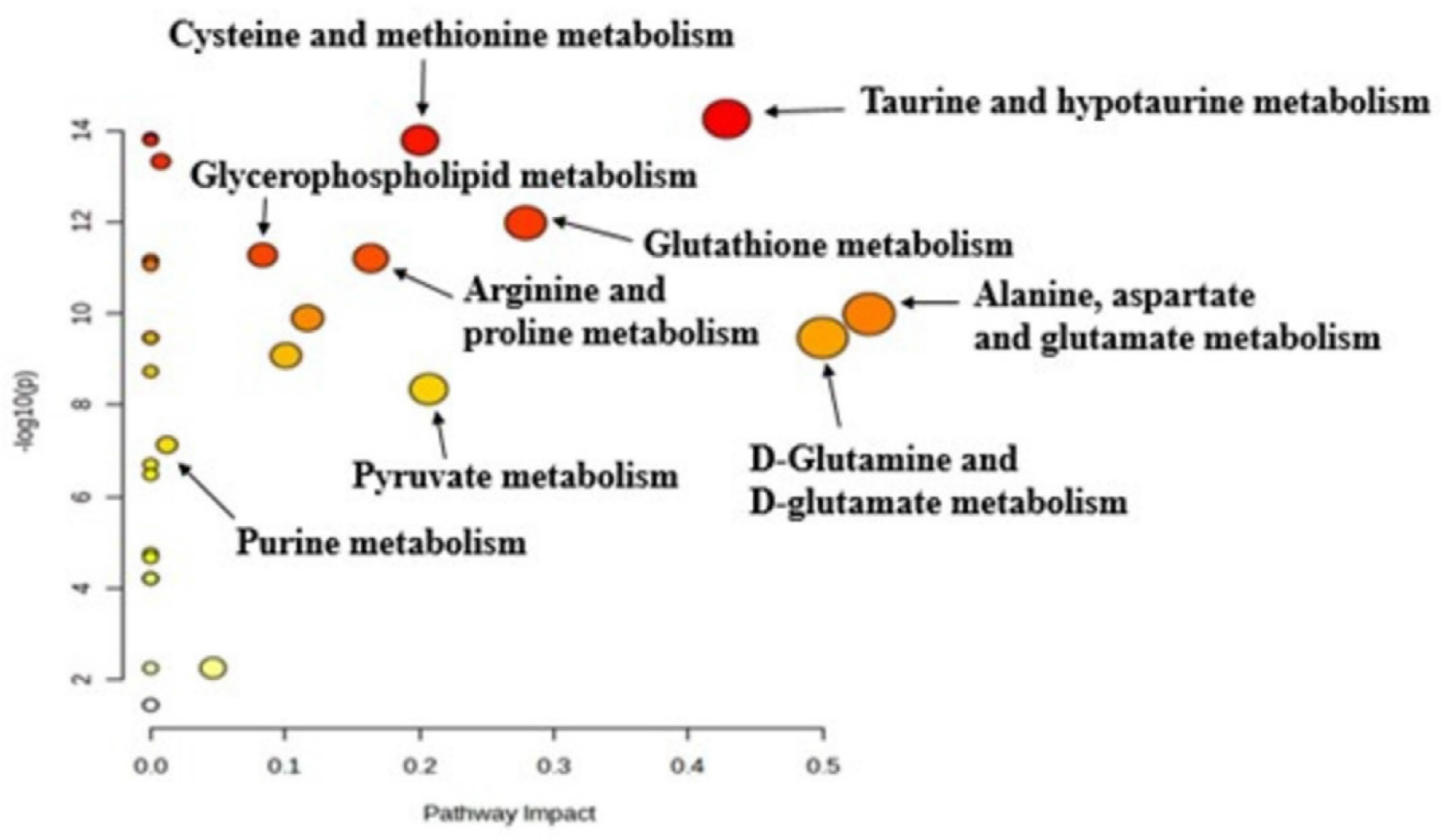
Pathway analysis generated with the MetaboAnalyst software between 4+1 and control groups for significant metabolites (p-value < 0.05), identifying the most relevant metabolic pathways. The color and size of each circle are based on the p-value and pathway impact value, respectively.

The pathway analysis tool integrated into the MetaboAnalyst software was used to identify altered metabolic pathways based on the most significantly altered metabolites in the 4+1 tamoxifen-resistant cell line compared to the control (sensitive) cell line. Pathway analysis revealed that cysteine, methionine, taurine, glutathione, proline, and purine metabolism were significantly altered in the 4+1 group compared to the control group **(Figure 4)**. The results of the metabolic pathway analysis are illustrated using a bubble plot, with each bubble representing a different metabolic pathway. The size of each bubble indicates the influence factor of the pathway.

### Metabolite profiling of the three different subtypes of treated groups

**Figure 5:**
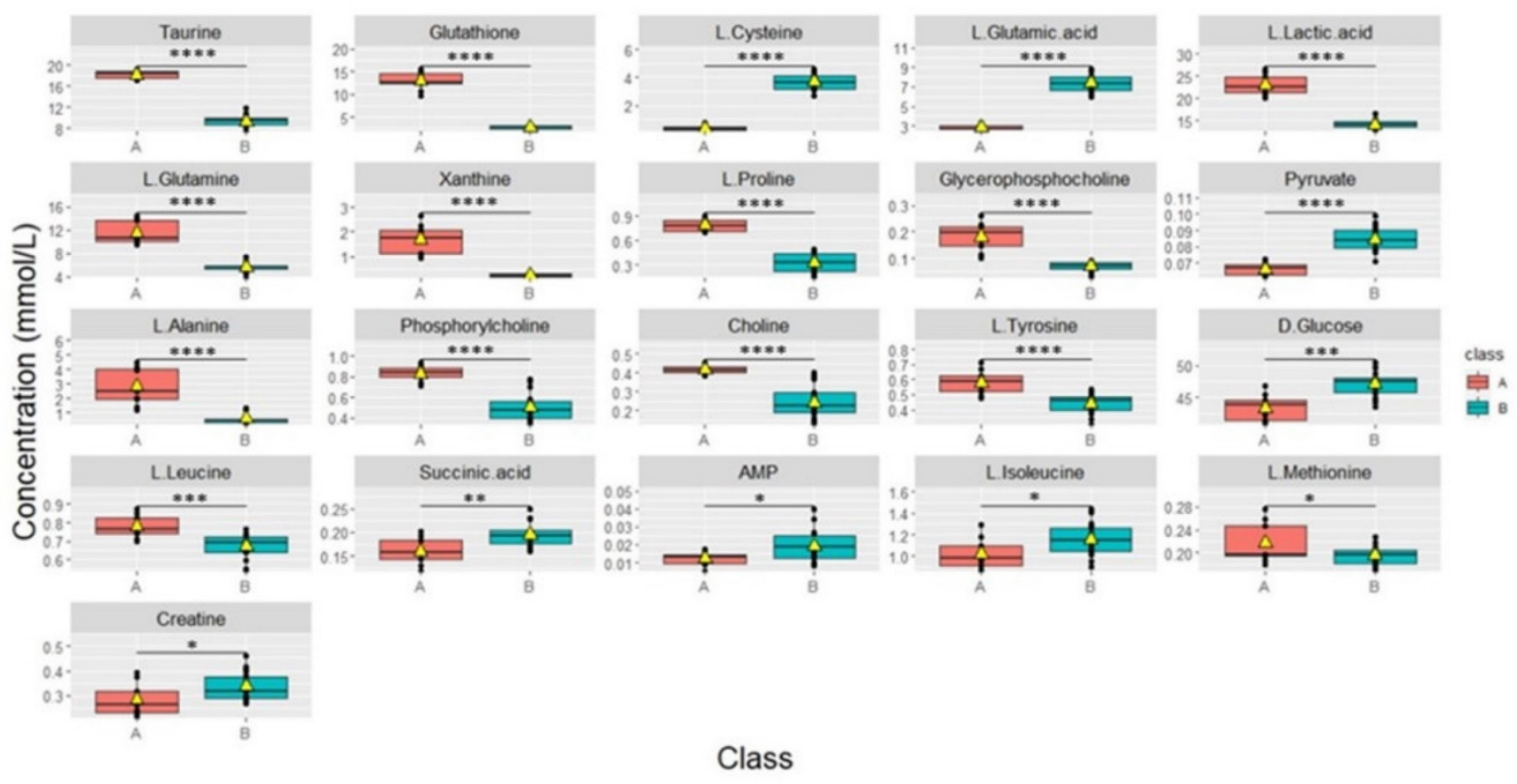
Boxplots of the concentrations of all significantly different metabolites between groups continuously treated with tamoxifen (n = 9) which was labeled (A) and group 50, 35x6 which was labeled (B) (n = 18). Data processing based on our raw data was performed by removing control samples and performing zero imputing. The black bar shows the median of a distribution, while the yellow triangle shows the average. Each box is drawn from the 25 to the 75 percentiles. p‐values of metabolites were determined with a Welch two-sample t‐test. *p < 0.05, **p< 0.01, ***p < 0.001, and **** *p* < 0.0001 calculated using the students' t-test.

Cells continuously treated with tamoxifen (4+1) exhibited significantly different changes in metabolite levels when compared to the cells in the other treated groups that received fixed concentrations of tamoxifen (50 and 35x6) for a specific duration of time, as described in the methods **(Figure 5).** “Area under the curve” (AUC) values were used to evaluate the ability of important metabolites to discriminate between groups. AUC was obtained from receiver operating characteristic (ROC) curves analysis based on the metabolite levels determined in the continuously treated group compared to 50 and 35x6 treated breast cancer cells (n = 27) analyzed in this study. Sixteen of the 41 metabolites showed an AUC value higher than 0.9, while nine of the 41 metabolites showed an AUC value equal to 1 **(Supplementary Table 2, Supplementary Figure 5)**. Based on their p-values **(Supplementary Table 3)**, taurine, glutathione, L-lactic acid, L-glutamine, xanthine, L-proline, glycerophosphocholine, L-alanine, phosphorylcholine, choline, L-tyrosine, L-leucine, and L-methionine significantly increased in group (A); whereas, L-cysteine, L-glutamic acid, pyruvate, D-glucose, succinic acid, AMP, L-isoleucine, and creatine significantly increased in group (B).

### Gene expression analysis

**Figure 6:**
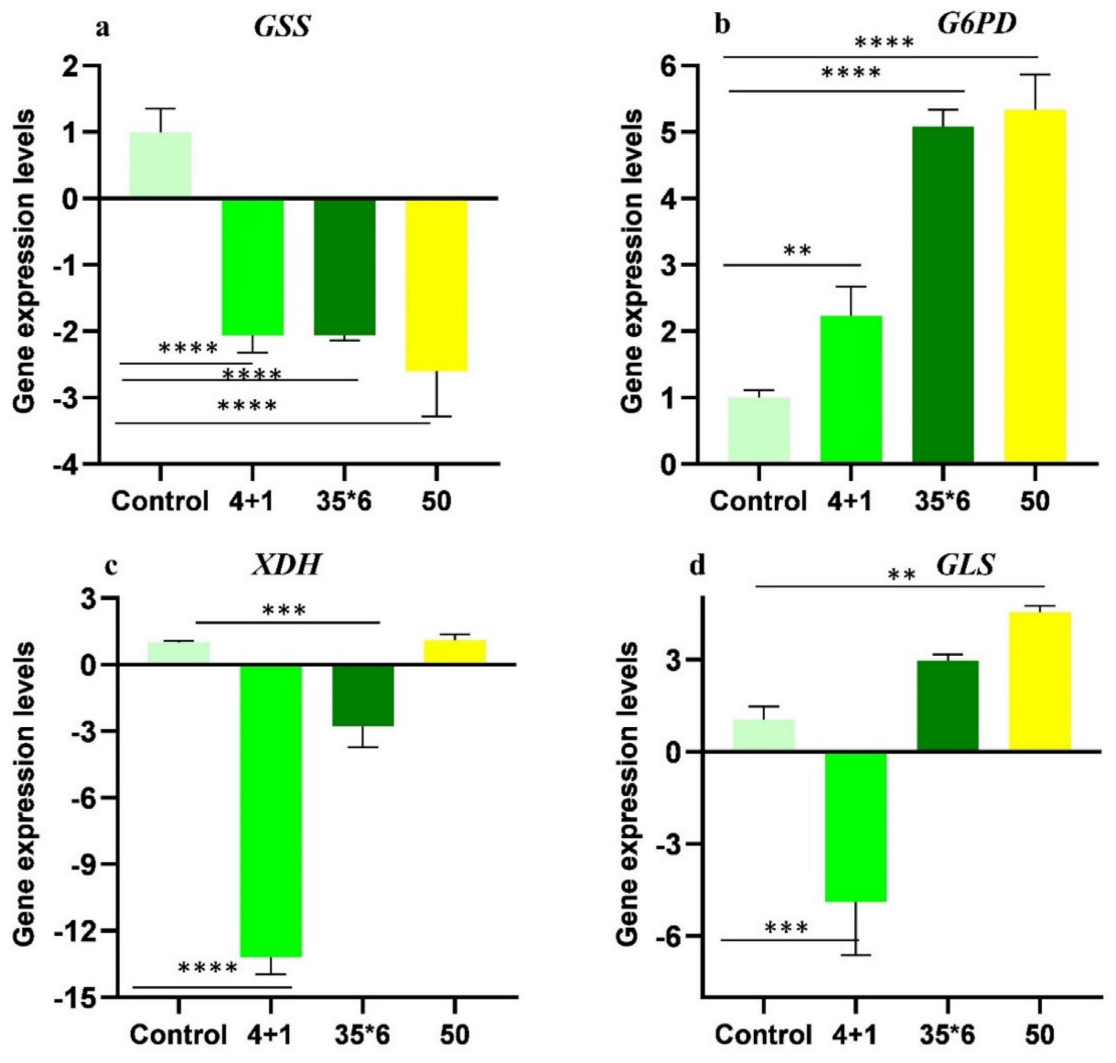
Relative gene expression levels (folds) of (A) *GSS*, (B) *G6PD*, (C) *XDH*, and (D) *GLS* of tamoxifen-resistant cell lines in comparison to control MCF-7 cell lines. Folds increase and decrease are presented ± SD and one-way ANOVA was used. p* < 0.05, p** < 0.01, p*** < 0.001, and p**** < 0.0001.

Metabolic profiling showed dysregulation of many metabolites with glutathione, xanthine, and glutamine exhibiting more significant differences among resistant cells maintained under tamoxifen (4+1) and the two other resistant cell lines (50 and 35x6). As a result, gene expression analysis of glutathione synthetase (*GSS*), glucose-6-phosphate dehydrogenase (*G6PD*), xanthine oxidoreductase (*XDH*), and glutaminase (*GLS*); the key enzymes involved in their metabolic pathways was carried out using RT-qPCR **(Figure 6)**. These specific enzymes were selected because they catalyze the committed steps in the synthesis or degradation of the respective metabolite. The results show that *GSS* was significantly downregulated by at least 2-fold in the three tamoxifen-resistant cell lines compared to the tamoxifen-sensitive control MCF-7 cells, while the expression of *G6PD* was significantly upregulated by 2- to 5-fold in all three treatment groups. In contrast, the expression of xanthine oxidoreductase (*XDH*) was significantly downregulated in cells maintained in 1 µM tamoxifen (4+1) as well as those treated six times with 35 µM tamoxifen (35x6) by 13- and 3-fold, respectively. The third tamoxifen-resistant cell line, which was treated with up to 50 µM tamoxifen showed no significant change in *XDH* expression. Meanwhile, the expression of *GLS*, which encodes the enzyme responsible for glutamate production from glutamine deamination, was significantly downregulated in the 4+1 treatment group by 5-fold but was increased in the 50 and 35x6 tamoxifen-resistance cell lines. Gene expression levels of *GLS* were correlated to the changes in the levels of glutamine and glutamate found in the three cell lines as seen in Figure 5.

**Figure 7:**
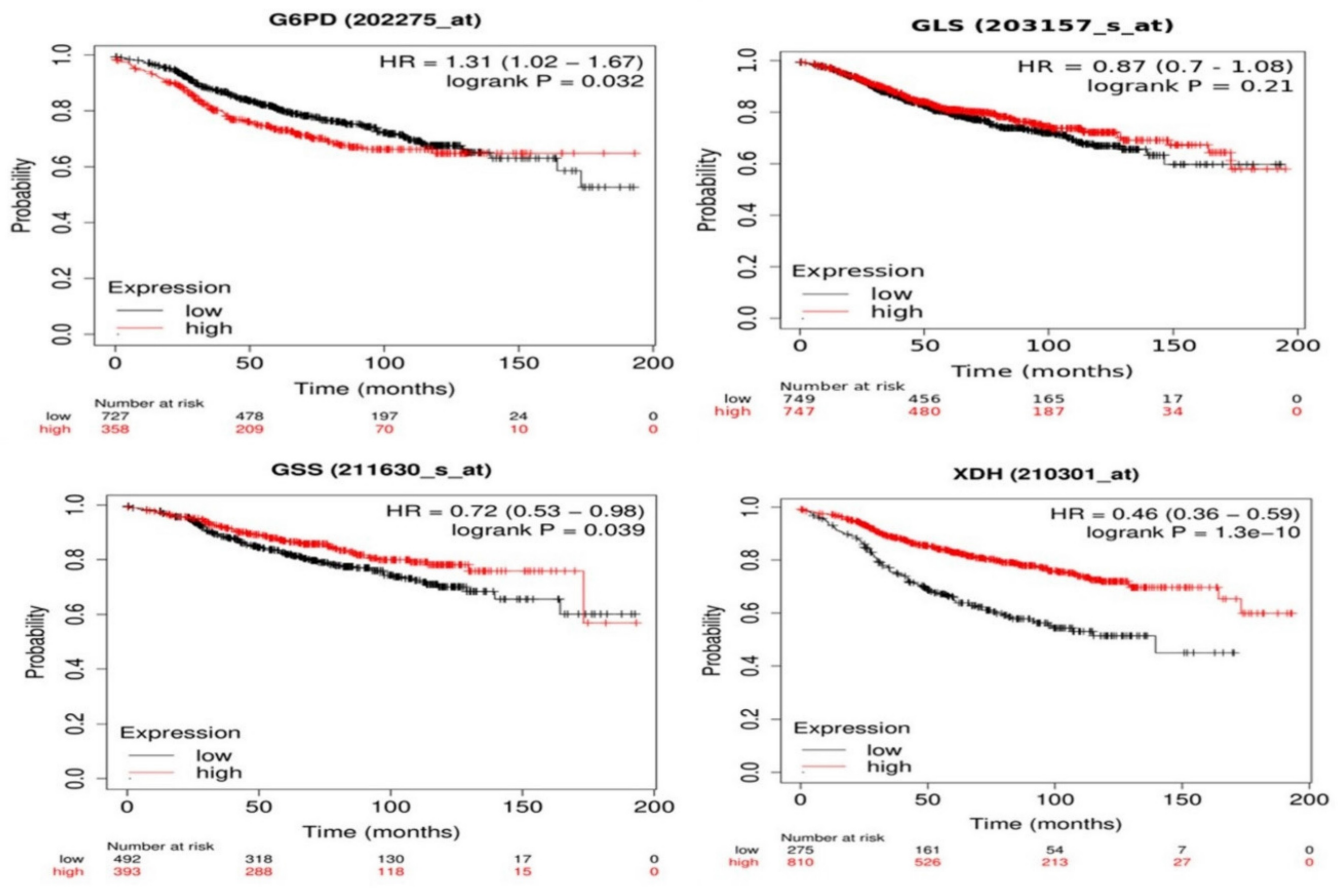
Kaplan-Meier survival curves of *G6PD, GLS, GSS*, and *XDH* with univariate Cox scores (p-values, (HR) hazard ratios) of BC patients treated with tamoxifen using Kaplan-Meier Plotter. GLS shows no significant link with the survival (p > 0.1). *G6PD* overexpression is linked to poor prognosis (HR > 1) while downregulation of *GSS* and *XDH* is significantly linked to poor prognosis and decreased overall survival (HR < 1).

Correlation between gene expression and overall survival of breast cancer patients receiving tamoxifen as endocrine therapy extracted from GEO and EGA repositories using Kaplan-Meier webtool (Kmplot.com) 32 showed that high expression of *G6PD* and low expression of *GSS* and *XDH* are significantly linked to poor prognosis and decreased overall survival **(Figure 7).** Changes in *GLS* gene expression are not significantly linked to overall survival among patients receiving tamoxifen and are included in the database. As seen in **Figure 7**, patients with low *G6PD* gene expression had a higher survival rate than patients with high *G6PD* expression (HR = 1.31, 1.02 - 1.67, p = 0.032). The median survival of the high expression group was 53.04 months while the median survival of the low expression group was 91.36 months (p = 0.032). Patients with low *GSS* and *XDH* expression had lower survival than patients with high expression levels (HR = 0.72, 0.53 - 0.98, p = 0.039) and (HR = 0.46, 0.36 - 0.59, p < 0.0001) for *GSS* and *XDH*, respectively. The median survival of the high-expression group of *GSS* was 173.2 months and the median survival of the low-expression group was 97.25 months (p = 0.039). Meanwhile, the median survival of the high-expression group of *XDH* was 107.43 months and the median survival of the low-expression group was 38 months (p < 0.0001). Overall, these data indicate that the expression of *G6PD*, *GSS*, and *XDH* could be further assessed to correlate tamoxifen treatment to patients' survival.

## Discussion

This study was designed to monitor the metabolic changes in tamoxifen resistance in MCF-7 breast cancer cell lines. There was an increase in glutamine, taurine, glutathione, and xanthine in the tamoxifen-resistant compared to sensitive cells. Furthermore, we observed a significant decrease in the branched-chain amino acids, valine and isoleucine, and a depletion in glutamate and cysteine levels. These metabolic changes were more prominent in cells under continuous tamoxifen treatment.

The increase in glutamine levels and the with decreased glutamate mainly in resistant cells maintained under tamoxifen indicates increased synthesis of glutamine from glutamate and decreased glutamine deamination. This was associated with increased expression of glutamate-ammonia ligase (*GLUL*) together and decreased *GSS* expression. This is in agreement with our previous study in which tamoxifen-resistant cells were reported to have high GLUL levels 22, which was attributed to the activation of the PI3K/AKT/PTEN signaling pathway, which is known to increase proliferation and cellular invasion 33. This was also reported in hepatic cells from mice treated with tamoxifen 34. Glutamate is a major source of carbon and nitrogen for the TCA cycle and transamination reactions, respectively. Additionally, it is used in the biosynthesis of glutathione 33, 35. Moreover, glutamine is an important source of amide nitrogen for nucleotide synthesis, and it contributes to the activation of the mTORC1 pathway which promotes mTORC1-dependant metabolic reprogramming, increasing the proliferation and growth of tumors 36-40.

In the current work, taurine was significantly increased in cells under continuous treatment of tamoxifen. Taurine is a nonproteinogenic amino acid that has protective and regulatory functions in many tissues, including the neural, cardiac, and skeletal muscle. It also has known direct and indirect antioxidant effects in normal tissues 41, and it downregulates the pro-apoptotic proteins in ischemic reperfusion injury 42, 43. Moreover, taurine interferes with mitochondria-dependent apoptosis and the unfolded protein response 44, it downregulates the p53-Chk1 pathway 45 and upregulates extracellular-signal-regulated kinases (ERK) and Wnt/β-catenin pathway 44, 46. The former increases survival by inhibiting apoptosis and increasing resistance to oxidative stress. Moreover, taurine has been shown to increase the expression of copper/zinc superoxide dismutase, catalase, and glutathione peroxidase (GPx) in B16F10 melanoma 47, and increase the activity of glutathione reductase in rats 48. The role of taurine in cancer is unclear and needs further studies, as there are conflicting studies on its role and effect in cancer and tumorigenesis, 49. There are studies demonstrating that taurine has pro-apoptotic effects on human colorectal cancer cells 50, inhibits lung metastasis in breast cancer in mice, and attenuates anthracene-induced breast tumorigenesis in mice 51, 52. On the other hand, increased levels of taurine were reported in retinoblastoma, glioblastoma, and medulloblastoma *in vivo* 53, 54.

Glutathione is an important scavenger of reactive oxygen species. It is either synthesized from γ-glutamyl-cysteine and glycine by glutathione synthetase (GSS) or recycled from glutathione disulfide by glutathione reductase (GSR) using NADPH as a reducing agent 33. Glutathione has a protective effect in normal tissues; 55 however, tumors also exploit this effect to protect themselves against oxidative stress. GSH has been associated with chemoresistance in many types of cancer, including breast, pancreatic, neuroblastoma, and glioblastoma 56-59. Cancers with higher GSH levels were more likely to survive vascular endothelial-induced oxidative and nitrosative stress and metastasize at different sites 60. These changes are caused by genetic and epigenetic changes in the tumor, and increased GSH was associated with epithelial-mesenchymal transition, local invasion, metastasis, and tumor survival 61, 62. Additionally, higher GSH concentrations were found in tumors exhibiting aggressive behavior compared to less aggressive tumors of the same kind or normal tissue 63. In breast cancer, increased GSH levels are associated with higher rates of metastasis, tumor growth, chemoresistance, and more aggressive behavior 64. Prolonged treatment of neuroblastoma cell lines with standard chemotherapy agents, etoposide or doxorubicin showed increased levels and synthesis of GSH, increased expression of γ-glutamyl-cysteinyl ligase, and decreased depletion of GSH 58, 65. Moreover, increased glutathione levels in glioblastoma were associated with higher rates of radiotherapy resistance when compared to cell lines with low GSH 66. The high levels of GSH, associated with the downregulation of GSS and increased G6PD expression, which is the main source of NADPH, is an indicator of a switch from GSH synthesis to glutathione recycling. This effect is more prominent in cells under continuous tamoxifen treatment.

The role of xanthine in resistance development is still not understood and more studies are needed to understand its role and the pathways associated with it, as it may be a potential marker for treatment resistance in different cancers, especially breast cancer. Xanthine is a purine base that is found in the body as a product of purine degradation. Xanthine is further oxidized by xanthine dehydrogenase (XDH) to form urate 67. However, xanthine could also be salvaged through the purine salvage pathway and used as a backbone for the synthesis of purine nucleosides, guanosine, and adenosine 68. Herein, an increase in xanthine levels and a decrease in *XDH* expression in cells under continuous tamoxifen treatment was noted indicating a switch from xanthine oxidation to the purine salvage pathway and thus increased purine nucleoside synthesis. Increased G6PD activity, which is the gatekeeping enzyme of the pentose phosphate pathway 69, and xanthine suggest an increased turnover rate for purine nucleosides, which may arise from the increased DNA damage from continuous exposure to tamoxifen. This hypothesis is supported by the fact that these changes are only observed in cells under continuous treatment and not the other cell lines. In another study, glioblastoma cell lines that had higher xanthine and hypoxanthine levels were more likely to develop radiotherapy resistance when compared to cell lines with lower xanthine levels 66. It was also previously shown that low XDH expression in breast cancer patients was associated with a lower disease-free survival (DFS) that was almost half the median survival time of patients with high XDH expression.

## Conclusion

Metabolic changes in tamoxifen-resistant MCF-7 cell lines were manifested with the increase of glutathione, xanthine, and other metabolites involved in antioxidant pathways along with their dysfunctional genes' expression. The significant increase in taurine, xanthine, and glutathione in tamoxifen-resistant cells indicates an increase in antioxidative activity in the cells, especially in the cell line under continuous tamoxifen treatment. A correlation of *XDH* and *GSS* genes' expression and poor prognosis among breast cancer patients treated with tamoxifen was seen. Further investigation of xanthine and glutathione's role in tamoxifen-resistant cells would highlight their prognostic value among BC patients.

## Supplementary Material

Supplementary scheme, figures and tables.

## Figures and Tables

**Table 1:** Metabolites (n = 41) detected in breast cancer cells treated with tamoxifen using three protocols to establish resistance (4+1, 50, 35x6) vs untreated cells using cryogenic probe NMR spectroscopy. Incidences represent the number of samples (9 samples in each group) in which the respective metabolite could be detected. Data processing was done as described in the methodological section. p‐values are used to compare metabolite levels in the different tamoxifen treatment groups to levels in untreated cells. p‐values for metabolites with significantly different levels between the two groups are shown in bold and were determined by performing a t‐test using Metaboanalyst software. *p < 0.05, **p< 0.01, ***p < 0.001, and ****p < 0.0001.

Metabolites	4+1	50	35x6
L-Cysteine	1.64E-14 ****	0.31573	0.37459
Taurine	4.756E-14 ****	0.36107	0.037432 *
Choline	1.89E-12 ****	0.0054549 **	2.211E-05 ****
L-Proline	4.836E-12 ****	0.24607	0.0011115 **
L-Glutamic acid	6.991E-12 ****	0.37563	0.55601
Glutathione	4.267E-11 ****	0.63693	0.2502
Phosphorylcholine	2.162E-10 ****	0.0087274 **	0.0005702 ***
L-Lactic acid	4.511E-09 ****	0.010465 *	0.0012575 **
L-Glutamine	1.992E-07 ****	0.71639	0.71033
Xanthine	1.048E-06 ****	0.49453	0.30562
D-Glucose	4.961E-06 ****	0.005362 **	0.010507 *
Glycerophosphocholine	1.771E-05 ****	0.78459	0.34956
L-Alanine	2.153E-05 ****	0.059341	0.025604 *
L-Isoleucine	6.168E-05 ****	0.0003494 ***	0.0021019 **
Pyruvate	0.0055735 **	0.388	0.22113
Creatine phosphate	0.027903 *	0.0001213 ***	7.261E-06 ****
L-Methionine	0.029121 *	0.67311	0.086321
L-Aspartic acid	0.03581 *	0.30317	0.014135 *
Tryptophan	0.050196	0.71498	0.025093 *
Histidine	0.11641	0.85935	0.081534
L-Tyrosine	0.13097	0.13079	0.0014601 **
Fumaric acid	0.18159	0.28003	0.79872
L-Arginine	0.18681	0.22732	0.75362
L-Phenylalanine	0.19337	0.41619	0.26398
Glycerol 3-phosphate	0.29278	0.066759	0.047568 *
L-Valine	0.33961	0.0027706 **	0.4856
Myo-Inositol	0.38766	0.33387	0.64249
L-Threonine	0.4278	0.0008473 ***	0.39498
L-Leucine	0.46269	0.013605 *	0.0017004 **
ATP	0.47863	0.35012	0.82597
Glycine	0.48907	0.36714	0.1938
Succinic acid	0.49288	0.019693 *	0.052117
Creatine	0.52339	0.20504	0.1217
AMP	0.53587	0.036564 *	0.60668
Acetic acid	0.5485	0.67169	0.93954
ADP	0.80683	0.87673	0.94498
L-Lysine	0.83533	0.40689	0.12504
NAD+	0.86299	0.82639	0.71918
NADH	0.91812	0.9737	0.35642
Serine	0.91853	0.41901	0.36719
Formic acid	0.97434	0.31877	0.11075
