# Supplementary material for: Glutathione and Xanthine Metabolic Changes in Tamoxifen Resistant Breast Cancer Cell Lines are Mediated by Down-Regulation of GSS and XDH and Correlated to Poor Prognosis
Source: J Cancer. 2024 May 30;15(13):4047–58. doi: 10.7150/jca.96659 (PMC11212086; doi:10.7150/jca.96659)
Supplement: Supplementary file 1 — Supplementary scheme, figures and tables. [file jcav15p4047s1.pdf]

Supplementary figures:

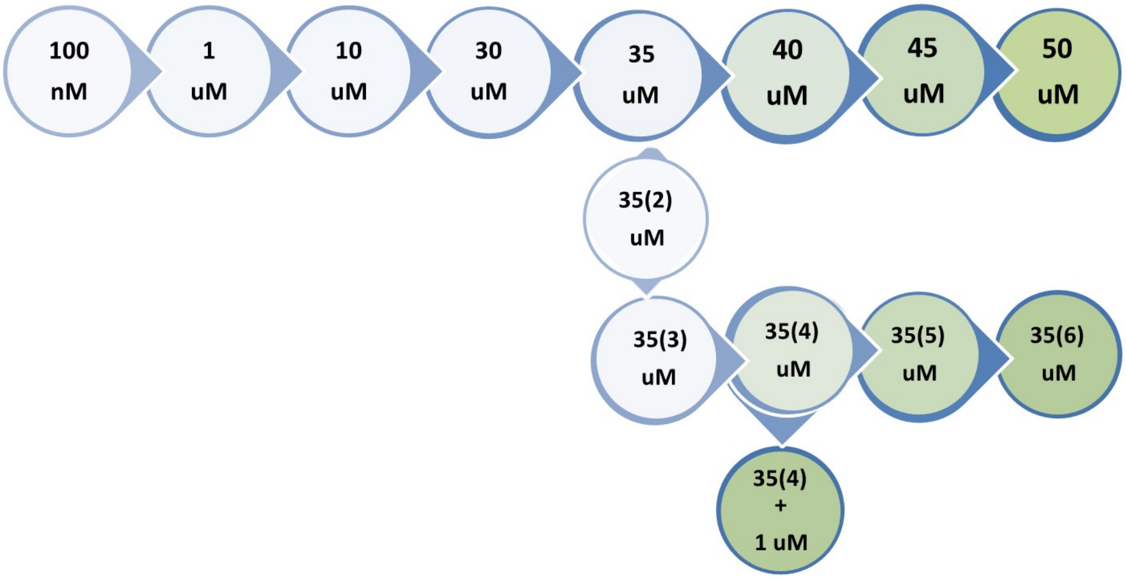

Scheme 1. Concentrations of tamoxifen used to treat sensitive MCF-7 cells to develop tamoxifen resistance to produce the three resistant cell lines.

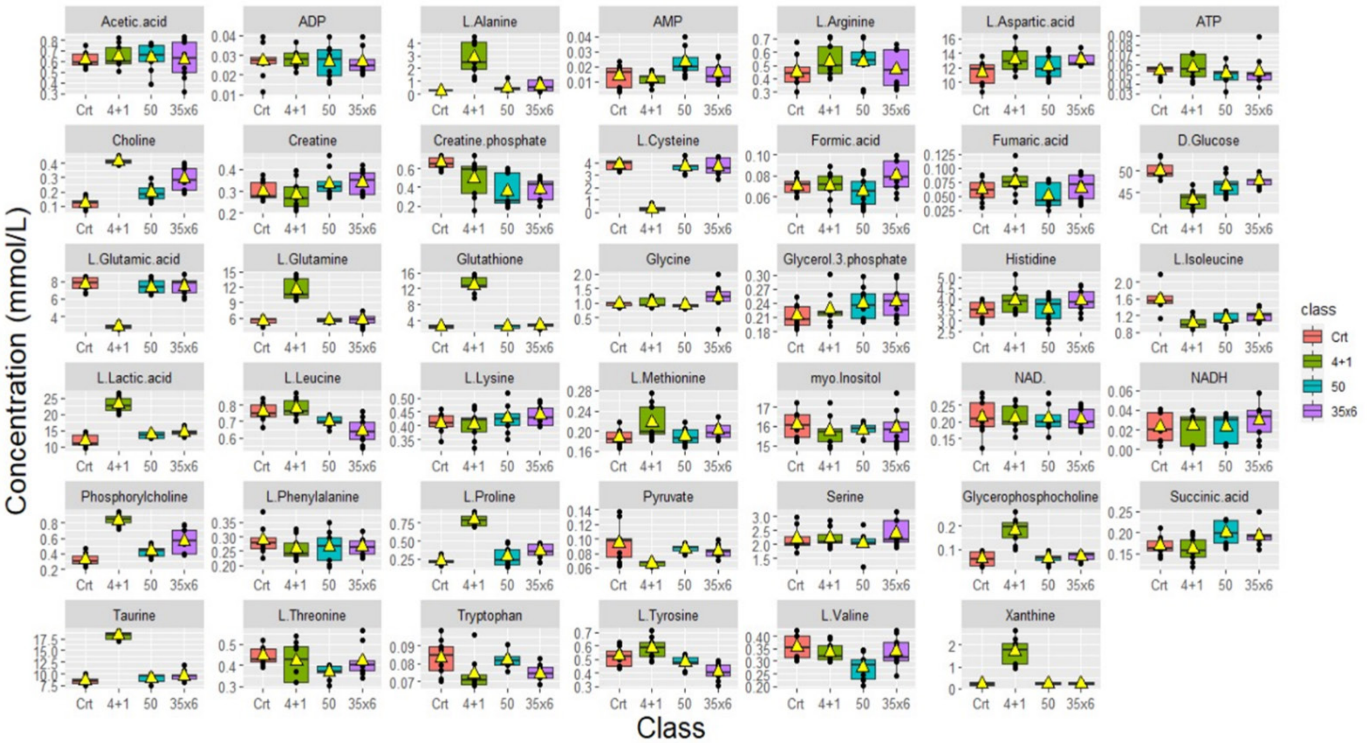

Figure S1. Metabolic changes in tamoxifen resistant MCF7 breast cancer cells. Boxplots showing the concentrations (y-axis) of the 41 metabolites via NMR-based profiling found in control (n = 9), 4+1 (n = 9), 50 (n = 9) and 35x6 (n = 9) groups. The black bars show the respective median of a distribution, while the yellow triangles show the respective average. Please note that the scale of the y-axis was adapted to the concentration range and is therefore different among the different metabolites.

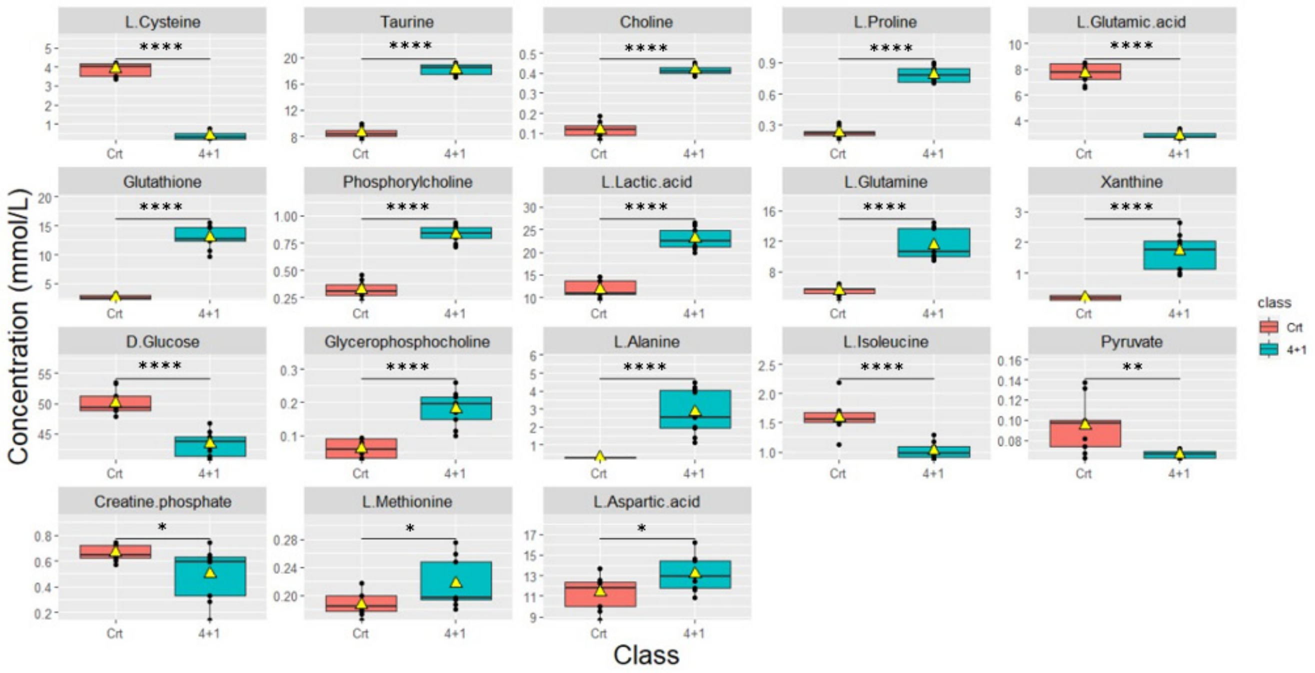

Figure S2. Metabolic changes in tamoxifen-resistant MCF7 breast cancer cells. Boxplots showing the concentrations (y-axis) of the significant metabolites found in control (n=9) vs 4+1 (n=9) groups. The black bars show the respective median of a distribution, while the yellow triangles show the respective average. Please note that the scale of the y-axis was adapted to the concentration range and is therefore different among the different metabolites. *p < 0.05, **p< 0.01, and ****p < 0.0001 calculated using the Students' t-test.

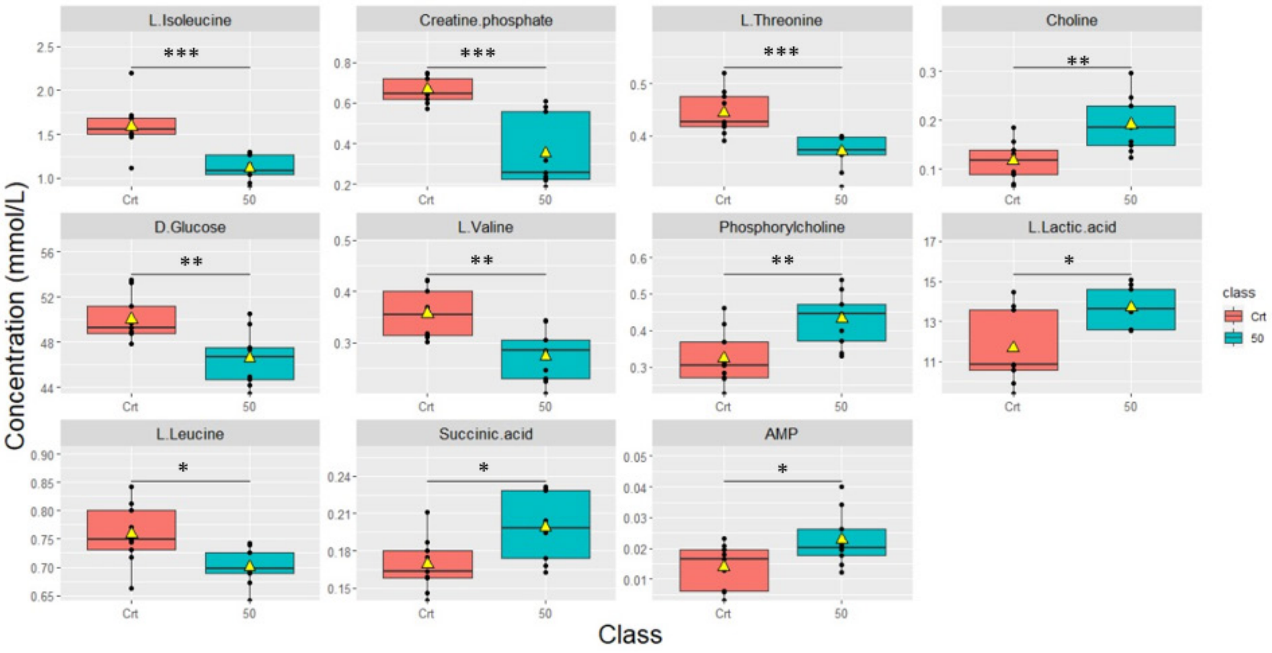

Figure S3. Metabolic changes in tamoxifen-resistant MCF7 breast cancer cells. Boxplots showing the concentrations (y-axis) of the significant metabolites found in control (n = 9) vs 50 (n = 9) groups. The black bars show the respective median of a distribution, while the yellow triangles show the respective average. Please note that the scale of the y-axis was adapted to the concentration range and is therefore different among the different metabolites. $*p < 0.05$, $**p < 0.01$, and $***p < 0.001$ calculated using the Students' t-test.

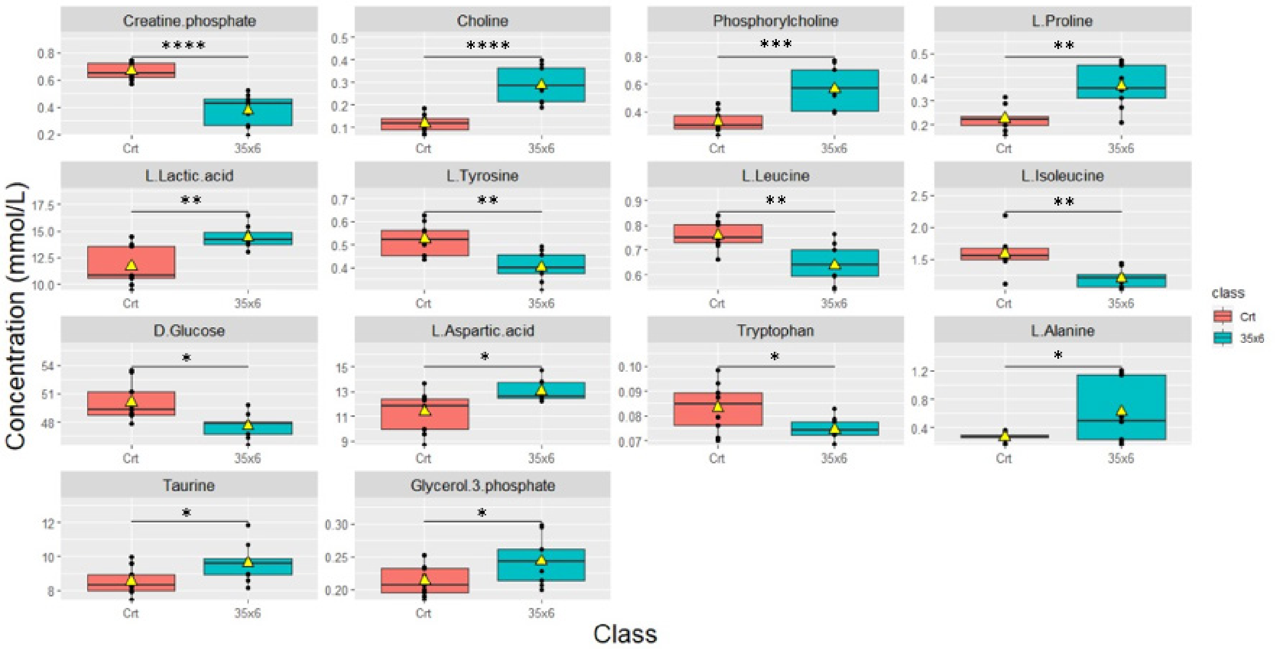

Figure S4. Metabolic changes in tamoxifen-resistant MCF7 breast cancer cells. Boxplots showing the concentrations (y-axis) of the significant metabolites found in control (n=9) vs 35x6 (n=9) groups. The black bars show the respective median of a distribution, while the yellow triangles show the respective average. Please note that the scale of the y-axis was adapted to the concentration range and is therefore different among the different metabolites. *$p < 0.05$, **$p < 0.01$, ***$p < 0.001$, and ****$p < 0.0001$ calculated using the Students' t-test.

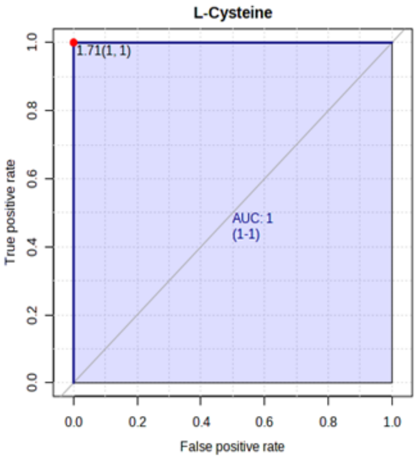
L-Cysteine
1.71(1, 1)
AUC: 1
(1-1)
True positive rate
False positive rate

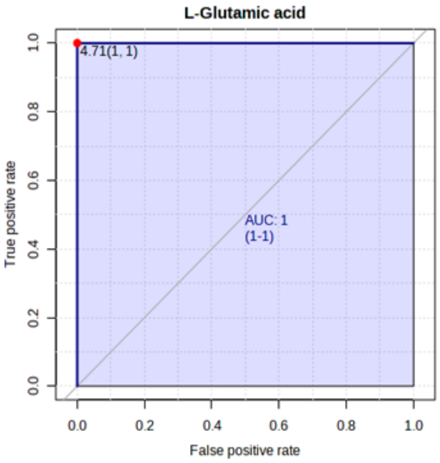
L-Glutamic acid
4.71(1, 1)
AUC: 1
(1-1)
True positive rate
False positive rate

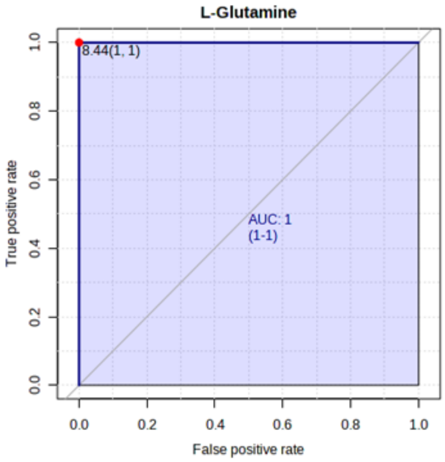
L-Glutamine
8.44(1, 1)
AUC: 1
(1-1)
True positive rate
False positive rate

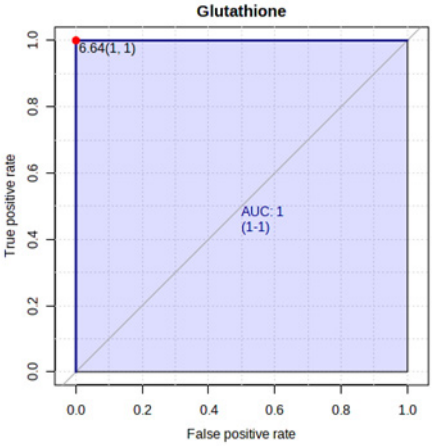
Glutathione
6.64(1, 1)
AUC: 1
(1-1)
True positive rate
False positive rate

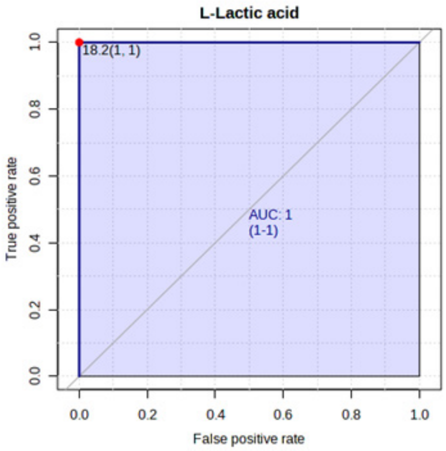
L-Lactic acid
18.2(1, 1)
AUC: 1
(1-1)
True positive rate
False positive rate

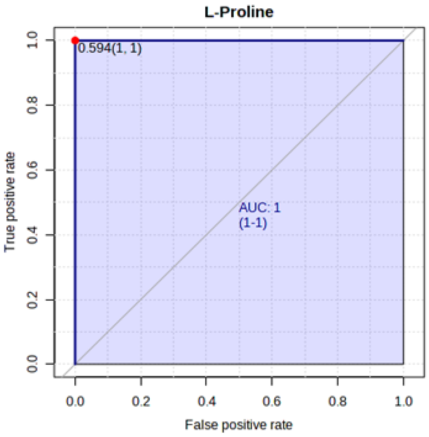
L-Proline
0.594(1, 1)
AUC: 1
(1-1)
True positive rate
False positive rate

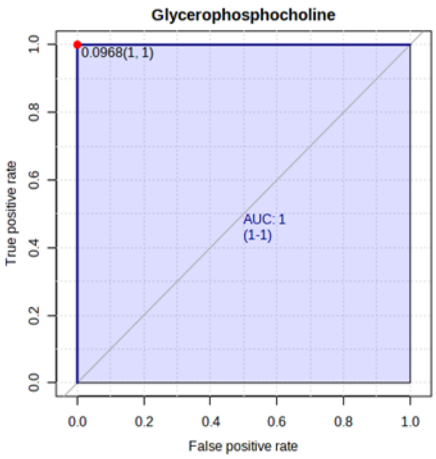
Glycerophosphocholine
0.0968(1, 1)
AUC: 1
(1-1)
True positive rate
False positive rate

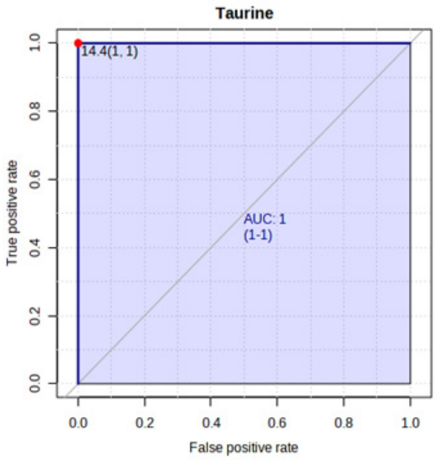
Taurine
14.4(1, 1)
AUC: 1
(1-1)
True positive rate
False positive rate

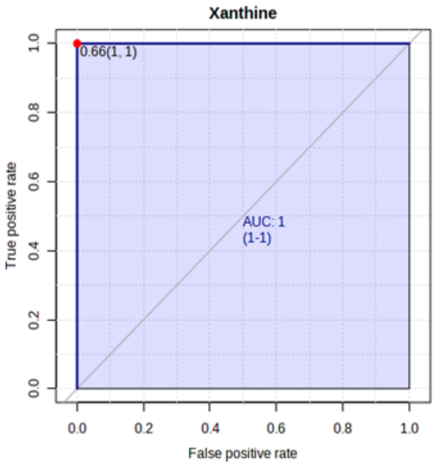
Xanthine
0.66(1, 1)
AUC: 1
(1-1)
True positive rate
False positive rate

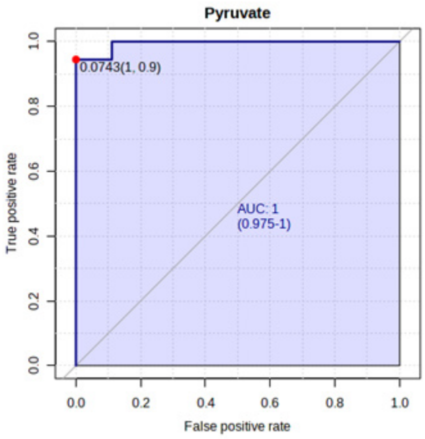
Pyruvate
0.0743(1, 0.9)
AUC: 1
(0.975-1)
True positive rate
False positive rate

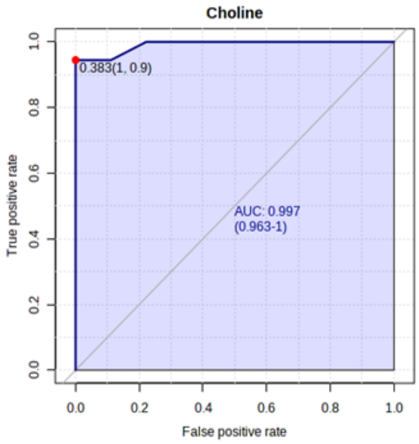
Choline
0.383(1, 0.9)
AUC: 0.997
(0.963-1)
True positive rate
False positive rate

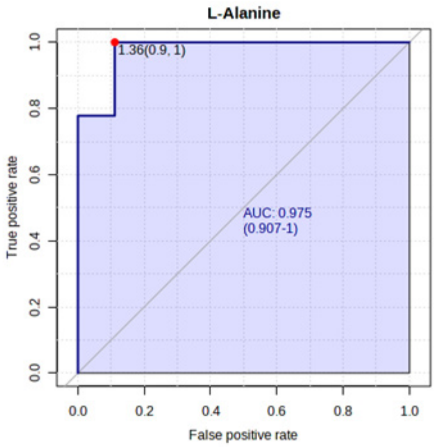
L-Alanine
1.36(0.9, 1)
AUC: 0.975
(0.907-1)
True positive rate
False positive rate

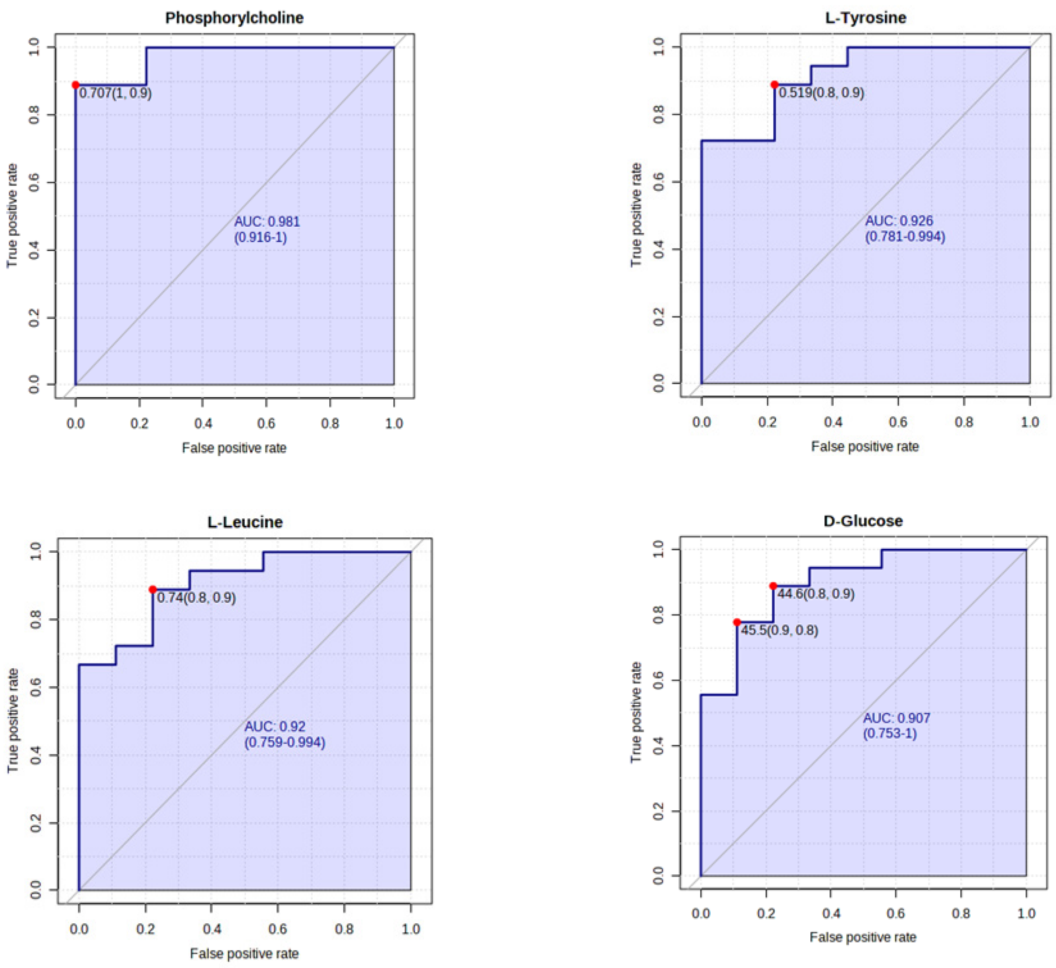

Figure S5. Receiver operating characteristic curves (ROC) are used to distinguish between groups 4+1 and 50, 35x6. The AUCs for 16 metabolites were above 0.9, indicating that they were of high diagnostic value.

Supplementary tables:

Table S1. Forward and reverse primers used in gene expression analysis.

Gene symbol	*Forward*	*Reverse*
**G6PD**	GGAAACGGTCGTACACTTCG	AGCCCACGATGAAGGTGTTT
**GLS**	CATCTTTGAAGGCCACTGCT	AGACCATGCTGAGAAAGCGT
**XDH**	ATGGGCCAAGGCCTTCATAC	TAGACGGCCTGTCCATTGAG
**GSS**	GAGAACCGTTCGCGGAGGAA	AGAGCGTGAATGGGGCATAG
**ACTB**	AGAGCTACGAGCTGCCTGAC	AGCACTGTGTTGGCGTACAG

Table S2. "Area under the curve" (AUC) values obtained from receiver operating characteristic (ROC) curves analysis in 4+1 compared to 50 and 35x6. based on the metabolite levels determined in the 27 treated breast cancer cells analyzed in this study. Nine of the 41 metabolites, namely L-cysteine, L-glutamic acid, L-glutamine, glutathione, L-lactic acid, L-proline, glycerophosphocholine, taurine, and xanthine, showed an AUC value equal to 1.

Metabolite	*AUC*	*Metabolite*	*AUC*	*Metabolite*	*AUC*
L-Cysteine	1	L-Leucine	0.90741	Myo-Inositol	0.58642
L-Glutamic acid	1	D-Glucose	0.90123	ADP	0.57716
L-Glutamine	1	Succinic acid	0.8179	Histidine	0.57407
Glutathione	1	AMP	0.75617	Glycine	0.5679
L-Lactic acid	1	Creatine	0.74691	L-Phenylalanine	0.5679
L-Proline	1	L-Isoleucine	0.74691	L-Aspartic acid	0.5679
Glycerophosphocholine	1	Tryptophan	0.74691	NADH	0.56173
Taurine	1	Creatine phosphate	0.73457	L-Arginine	0.55864
Xanthine	1	Fumaric acid	0.70062	L-Threonine	0.55556
Pyruvate	0.99383	L-Valine	0.67901	Formic acid	0.53704
Choline	0.99074	L-Methionine	0.66667	NAD+	0.51852
L-Alanine	0.97531	ATP	0.64198	Acetic acid	0.51235
Phosphorylcholine	0.97531	L-Lysine	0.6358	Serine	0.50617
L-Tyrosine	0.91975	Glycerol 3-phosphate	0.62346		

Table S3. Metabolites (n = 41) detected in breast cancer cells continuously treated samples with tamoxifen (A) vs 50, 35x6 (B) using cryogenic probe NMR spectroscopy. Given are p-values. Incidences represent the number of samples (continuously treated (n=9) and group 50, 35x6 (n=18)), in which the respective metabolite could be detected. As data processing for statistical evaluation described in methodological part, p-values study the comparison of metabolite levels in the group of continuously treated cells (A) and group 50, 35x6 (B). Metabolites (n = 21) with significantly different levels between the two groups are shown in bold. p-values were determined by t-test using Metaboanalyst.

Metabolites	*P-value*	*Metabolites*	*P-value*	*Metabolites*	*P-value*
**Taurine**	2.12E-17	**D-Glucose**	0.000101	L-Aspartic acid	0.37291
**Glutathione**	2.41E-17	**L-Leucine**	0.000336	L-Threonine	0.42763
**L-Cysteine**	4.69E-15	**Succinic acid**	0.00324	Histidine	0.46724
**L-Glutamic acid**	2.11E-14	**AMP**	0.029101	L-Arginine	0.57871
**L-Lactic acid**	3.64E-13	**L-Isoleucine**	0.035111	ADP	0.64457
**L-Glutamine**	3.40E-11	**L-Methionine**	0.040434	NADH	0.65593
**Xanthine**	7.65E-11	**Creatine**	0.040595	Glycine	0.67438
**L-Proline**	9.88E-11	Fumaric acid	0.059914	L-Phenylalanine	0.67818
**Glycerophosphocholine**	1.63E-08	Creatine phosphate	0.061416	Formic acid	0.71988
**Pyruvate**	2.27E-07	Tryptophan	0.1523	Acetic acid	0.73349
**L-Alanine**	2.60E-07	L-Lysine	0.16593	myo-Inositol	0.76999
**Phosphorylcholine**	4.31E-07	L-Valine	0.19795	NAD+	0.87593
**Choline**	2.00E-06	ATP	0.28395	Serine	0.98869
**L-Tyrosine**	6.69E-05	Glycerol 3-phosphate	0.31052		
